# Histological Chorioamnionitis Is Increased at Extremes of Gestation in Stillbirth: A Population-Based Study

**DOI:** 10.1155/2011/456728

**Published:** 2011-07-19

**Authors:** Adrienne Gordon, Monica Lahra, Camille Raynes-Greenow, Heather Jeffery

**Affiliations:** ^1^Department of Neonatal Medicine, Royal Prince Alfred Hospital, Sydney, NSW 2050, Australia; ^2^Sydney School of Public Health, University of Sydney, Sydney, NSW 2006, Australia

## Abstract

*Objective*. To determine the incidence of histological chorioamnionitis and a fetal response in stillbirths in New South Wales (NSW), and to examine any relationship of fetal response to spontaneous onset of labour and to unexplained antepartum death. *Study Design*. Population-based cohort study. *Setting*. New South Wales Australia. *Population*. All births between 2002 and 2004 with stillbirths reviewed and classified by the state perinatal mortality review committee. *Methods*. Record linkage of the Midwives Data Collection and the Perinatal Death Database including placental histopathology and standardised cause of death classification. *Results*. 952 stillbirths were included. The incidence of histopathological chorioamnionitis was 22.6%, with a bimodal distribution. A fetal inflammatory response was present in 10.1% and significantly correlated with spontaneous onset of labour. The absence of a fetal inflammatory response was strongly associated with unexplained antepartum death. *Conclusions*. The increased incidence of histological chorioamnionitis at extremes of gestation is confirmed in the largest dataset to date using population data. This has important implications for late gestation stillbirth as the percentage of unexplained stillbirths increases near term.

## 1. Introduction

Stillbirth remains a major unresolved public health issue and is identified globally as a priority area for targeting prevention strategies in perinatal health. Many deaths remain unexplained despite investigation, and there is an urgent need to identify and target appropriate areas for research and prevention of stillbirth. 

In developed country settings with low neonatal mortality rates, stillbirths now comprise the majority of perinatal deaths with no significant change in rates over the past 20 years [1]. There is even evidence of a recent increase in stillbirth rates in Australia, UK, and USA which has been attributed to an increase in population risk factors such as advanced maternal age, nulliparity, and obesity [2, 3]. In Australia, stillbirths account for around two thirds of all perinatal deaths with a reported rate of 7.4 per 1000 births in 2007 [1]. Many of these deaths remain unexplained with proportions documented as 41% of stillbirths in NSW and 28% nationally [4]. Stillbirths nearer to term are more likely to be classified as unexplained than very preterm stillbirths [5]. We recently reported that in NSW a striking 60% of term stillbirths were unexplained [4]. A number of these “unexplained” deaths equate to “unexplored” with the proportion of unexplained deaths shown to be lower in settings with an extensive test protocol and higher autopsy rates [6, 7]. 

Infection has been documented as particularly important in early stillbirths with a strong association between intrauterine infection and births before 28 weeks [8]. Intrauterine infection is caused by ascending infection from the lower genital tract [9], is usually asymptomatic until labour onset, and the gold standard diagnosis is most commonly retrospective after histopathologic examination of the placenta [10]. Ascending intrauterine infection elicits both maternal (chorioamnionitis) and fetal inflammatory responses (chorionic and umbilical vasculitis, funisitis) [8]. Recent data from a hospital cohort of 459 stillbirths has shown a rise in incidence of chorioamnionitis in later gestation stillbirths suggesting that some late unexplained stillbirths may be due to infection [10]. Intrauterine infection is a recognized cause of stillbirth, and it is postulated that undiagnosed infection may account for a proportion of unexplained deaths [10, 11].

Assessing the relationship between intrauterine infection, inflammation, and stillbirth on a comparative basis is difficult, as there are different methods of reporting on the pathological findings and no international standardised criteria. There are also widely differing gestational ages after which an intrauterine death is defined as a stillbirth with countries such as Sweden until recently defining stillbirth as death beyond 28 weeks gestation compared with the 20 week gestation definition of Australia. Both these differences likely explain why the incidence of chorioamnionitis in the literature is variably reported from <10 to >90% [11–28]. There are considerably fewer studies that report on the incidence of a fetal inflammatory response despite accumulating evidence indicating that this is the histopathology most correlated with outcomes related to intrauterine infection [14–18]. 

The majority of earlier published studies of stillbirth and chorioamnionitis are small and comprise case series, case-control studies, or hospital-based cohorts [12, 13, 19–28]. There are no large population-based studies utilising record linkage of histological chorioamnionitis and stillbirth. 

We have shown recently, in the largest published hospital cohort of placentae of stillborn infants, an overall incidence of chorioamnionitis of 36% and demonstrated the novel finding of increased incidence at extremes of gestational age, with >60% of placentae showing chorioamnionitis at both 22 and 41 weeks. [10]. We also found that a fetal inflammatory response was strongly associated with spontaneous onset of labour and its absence with unexplained antepartum death. These findings support the “failure to rescue by birth” hypothesis. This hypothesis suggests that the fetal immune system responds to intrauterine infection by a sequence which leads to early labour and that a fetus who dies in utero in the presence of histological chorioamnionitis may have been unable to mount an immune response large enough to trigger the onset of labour [11, 29].

The purpose of this study was to determine whether our novel findings could be replicated in a large population based cohort with placental pathology performed in different centres.

## 2. Methods

This was a Statewide population-based cohort study using deidentified linked data from two NSW data sets: the New South Wales Midwives Data Collection and the Perinatal Death Data from the NSW Maternal and Perinatal Committee. Placental pathology was performed by each area referral pathology service, and reports were scanned and linked to the dataset. Ethical approval was obtained from the NSW Department of Health Ethics Committee Ref No DoHEC 2005-06-11. 

### 2.1. Data Sources

The New South Wales Midwives Data Collection (MDC) is a population-based surveillance system covering all births in NSW public and private hospitals, as well as home births [30]. It encompasses all live births and stillbirths of at least 20 weeks gestation or at least 400 grams birth weight. The MDC requires the attending midwife or doctor to complete a notification form when a birth occurs, and collects demographic, maternal health, pregnancy, labour, delivery, and perinatal outcome data (see Appendix S1 in Supplementary Material available online at doi:10.1155/2011/456728). 

The NSW Maternal and Perinatal Committee is a quality assurance committee established under the NSW Health Administration Act 1982 and is privileged under this Act to carry out confidential reviews of both maternal and perinatal deaths [30]. Members are appointed by the Minister for Health. A subgroup called the Perinatal Outcomes Working Party (POWP) reviews and classifies perinatal deaths using the Perinatal Society of Australia and New Zealand (PSANZ)—Perinatal Mortality Classification System which has been documented to have a high interobserver reliability with a kappa value of 0.83–0.95 [31, 32]. Information available to the POWP at review is forwarded by hospitals and includes a confidential report on perinatal death (Appendix S2), postmortem, and placental pathology reports as well as any other information considered relevant by the local hospital perinatal death review committee. Information considered by the Committee is confidential. 

### 2.2. Placental Examination

Guidelines for placental reporting are included in the PSANZ Guidelines on Perinatal Mortality Audit [32]. Placental examinations were performed by an Anatomical Pathologist at the local pathology service where the stillbirth occurred and reports forwarded to the Perinatal Outcomes Working Party. For this study, all the placental reports were then further reviewed by one researcher (AG) for the presence of chorioamnionitis and a fetal inflammatory response.

Chorioamnionitis was defined as linear aggregation along tissue planes of neutrophil polymorphs of maternal origin in the subchorionic fibrin, chorion or amnion of the peripheral membranes, or the fetal plate of the placenta. Fetal Inflammatory response was defined as the presence of umbilical vasculitis and/or funisitis or inflammation of the chorionic plate. Umbilical vasculitis was defined as migration of fetal neutrophil polymorphs into or through the media of the umbilical arteries or vein, usually in the direction of the amniotic surface of the cord. Funisitis was defined as further migration of fetal neutrophil polymorphs into the Wharton's jelly of the umbilical cord. 

### 2.3. Study Population

Records for babies from the Midwives Data Collection and Perinatal Deaths data compiled by the NSW Maternal and Perinatal Committee subgroup POWP were linked using probabilistic record linkage methods in Automatch software (MatchWare Technologies Inc, Silver Spring Md, USA). Both datasets covered the years 2002–2004. Babies were included if they had no congenital abnormality and had placental histopathology performed. Cause of death classification was extracted from the perinatal death database on all stillborn infants that had been reviewed by the Perinatal Outcomes Working Party. Demographic data were collected from both datasets. 

### 2.4. Definitions

Gestational age was determined by certain dates confirmed by ultrasound before 20 weeks gestation or if dates uncertain by ultrasound alone prior to 20 weeks or if unavailable by examination of the newborn infant.

Stillbirth was defined as a baby born of at least 20 weeks gestation or 400 g birth weight who did not, at any time after delivery, breathe or show any evidence of life such as a heartbeat.

Spontaneous labour was defined by the initiation of regular painful uterine contractions without medical or surgical intervention.

Unexplained antepartum death was defined as the death of a normally formed fetus prior to the onset of labour where no predisposing factors are considered likely to have caused the death. 

### 2.5. Statistical Analysis

Statistical analysis was performed using SPSS (Statistical Package for Social Sciences) version 14.0. Independent proportions were compared using the chi-squared test or Fishers exact where appropriate. Multivariate logistic regression was performed to assess the relationship of fetal inflammatory response with labour and unexplained death adjusting for confounders. Curve fit analysis using least squares regression fitting was performed on the distribution of incidence of chorioamnionitis using Igor Pro software (version 5.02; Wavemetrics, Inc, Lake Oswego, OR, USA). Best fit model was ascertained by the minimisation of the chi-square of the residuals. 

## 3. Results

There were a total of 258 045 births in NSW over the three-year study period. The total number of perinatal deaths reviewed and classified by the Perinatal Outcomes Working Party was 1877, and of these, there were 1264 stillbirths and 613 neonatal deaths. Of the 1264 stillbirths, 1096 stillbirths (87%) had no congenital abnormality, and 952 (87%) had placental pathology performed and were included in the cohort. For 95 % of the cohort (901/952), there was additional descriptive data available from the Midwives Data Collection. Demographic data is shown in Table 1.

### 3.1. Incidence of Histological Chorioamnionitis

**Figure 1:**
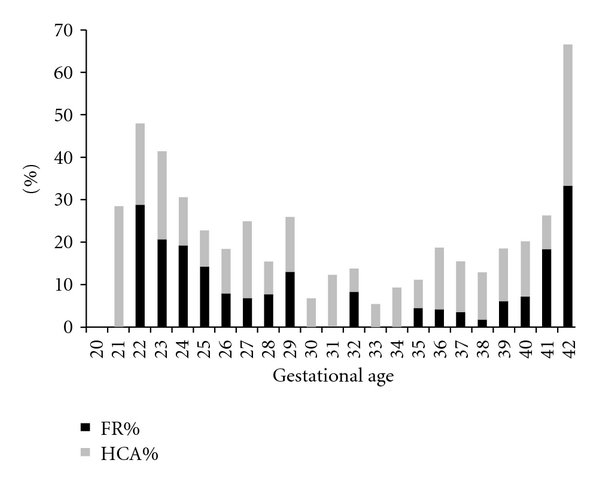
Histological chorioamnionitis and fetal response in stillbirths by gestational age in NSW 2002–2004 (*N* = 952).

Histological chorioamnionitis without a fetal response was present in 12.5% (119/952), and histological chorioamnionitis with a fetal response was present in 10.1% (96/952). No babies had a fetal response without histological evidence of chorioamnionitis. Overall, the incidence of histological chorioamnionitis was 22.6% (215/952). The distribution of chorioamnionitis by completed week of gestation is shown in Figure 1. The incidence was increased at the extremes of gestation, and curve fit analysis by the least squares method showed that residual values were lowest for a bimodal Gaussian distribution. This distribution remained the best fit when a sensitivity analysis was performed without the 3 babies born at 42 weeks. 

### 3.2. Relationship of Fetal Response to Labour Onset

There was a significant association between spontaneous onset of labour and the presence of a fetal inflammatory response. The proportion of stillborn babies who had a fetal inflammatory response whose mothers had spontaneous onset of labour was 67% (65/96) significantly higher than the 27% (238/856) with no fetal inflammatory response. Adjusted odds ratios are shown in Table 2. 

### 3.3. Absence of Fetal Response and Unexplained Death

The absence of a fetal inflammatory response was strongly associated with the classification of unexplained antepartum death. The proportion of stillborn babies without a fetal inflammatory response who were classified as unexplained deaths was 53% (454/856) compared with only 16% classified as unexplained with a fetal inflammatory response. Adjusted odds ratios are shown in Table 2. The association of a fetal inflammatory response with the other PSANZ classifications for the cause of death is documented in Table 3. 

## 4. Discussion

This represents the largest study of histological chorioamnionitis in a stillborn cohort to date and is truly population based. We have confirmed our previously published findings of a bimodal distribution of chorioamnionitis in stillbirth with increased incidence in both early and late gestation. We have also reproduced the striking correlation between the presence of a fetal inflammatory response and the spontaneous onset of labour, and the absence of that fetal inflammatory response and the classification of unexplained antepartum death using a standardised classification system. 

The strengths of this study are that it is population based and, therefore, includes a large number of stillbirths from a birth cohort of more than 250,000 women reducing the likelihood that our findings may have occurred by chance. With a larger dataset, we were able to perform multivariate analysis to test the associations of labour and unexplained death with fetal inflammatory response and adjust for potential confounders. This represents further contribution to the area as the majority of previous studies have provided univariate data only [12, 13, 19–28]. 

The classification of cause of death uses the PSANZ Perinatal Death Classification system which has been endorsed nationally and has now also been shown to perform well against other classification systems used internationally [33]. The members of the committee who attribute the classification all have clinical content knowledge, relevant investigations, and confidential reports forwarded from the hospital of delivery and are experienced in classifying perinatal deaths. 

There are, however, inherent weaknesses in using large population-based datasets. The detail that can be obtained is limited to what routine information is collected and there is often a significant time lag between when the information is collected to when it is published or available to researchers. Placental pathology was performed in different laboratories and by different pathologists; however, previous literature has shown that even with different pathologists intra- and interobserver agreement of the presence of intrauterine inflammation remains consistently high as does the clustering of findings that relate to ascending infection [34]. Furthermore, all reports were reviewed and coded by one clinical researcher.

A further issue as in many studies that assess stillbirth is the unavailability within the same cohort of placental pathology on live born infants. It is well documented that there is a high incidence of chorioamnionitis in preterm live born babies that decreases with gestation [35]. There is minimal data in term live born babies but what is published suggests this incidence is low. Previous studies that have assessed chorioamnionitis in later gestation in live born babies have shown a slight increase in healthy term infants of 3.6% at term and 5.1 % after term [36]. A more recent study that assessed fetal inflammatory response at term showed an incidence of 4% and associated this with both microbial invasion of the amniotic cavity and intra-amniotic inflammation [37]. Both these indicate a significantly lower incidence at term than that reported in stillbirths in both this cohort and in our previous hospital cohort.

In summary, we confirm our previous findings of a bimodal distribution of histological chorioamnionitis in stillbirth. This consistent finding of an increased incidence of histological chorioamnionitis in term stillborn infants is important and requires further exploration. On average, 20–70% of stillbirths are unexplained and the unexplained group increases with gestation with 60% of NSW stillbirths remaining unexplained at 37 weeks or greater [4]. Our findings suggest that undiagnosed infection may be implicated in a proportion of these unexplained deaths. We have also been able to demonstrate clearly, adjusting for confounders, the significant relationships between spontaneous onset of labour and fetal response and the absence of that fetal response and the classification of unexplained antepartum death. This contributes to the “failure to rescue by birth” hypothesis that a fetus who is unable to mount a sufficient immune response to trigger labour may be more likely to die in utero [11, 29].

Future research in this area requires elucidation of the underlying biological mechanisms of intrauterine inflammation and its consequences, further study of histological chorioamnionitis in term infants, and standardisation of placental reporting. Priority areas related to underlying mechanisms include molecular and genetic studies assessing fetal and maternal immune function. The incidence and significance of histological chorioamnionitis needs to be determined for term live born infants within large cohorts where antenatal, delivery, and outcome data are known. Finally, the development of an international standardized approach to placental pathological examination and reporting is long overdue and will enable comparison of these findings in different settings. 

## Supplementary Material

Appendix S1: The New South Wales Midwives Data Collection (MDC) is a population-based surveillance system covering all births in NSW public and private hospitals, as well as home births. It encompasses all live births and stillbirths of at least 20 weeks gestation or at least 400 grams birth weight.
Appendix S2: The NSW Maternal and Perinatal Committee is a quality assurance committee established under the NSW Health Administration Act 1982 and is privileged under this Act to carry out confidential reviews of both maternal and perinatal deaths. Information available to the POWP at review is forwarded by hospitals and includes a confidential report on perinatal death (Appendix S2), postmortem, and placental pathology reports as well as any other information considered relevant by the local hospital perinatal death review committee.

## Figures and Tables

**Table 1:** Demographic details of stillborn cohort.

Variable	*n* = number	Percentage (*n/N*)
*Maternal age group (year s)		
< 20	44	4.9
20–24	143	15.9
25–29	246	27.3
30–34	275	30.5
35–39	148	16.4
≥40	45	4.8
*Primigravid		
Yes	423	46.9
No	478	53.1
*Preexisting diabetes		
Yes	16	1.8
No	885	98.2
*Gestational diabetes		
Yes	27	3
No	874	97
Chronic hypertension		
Yes	29	3
No	923	97
Preeclampsia		
Yes	59	6.2
No	893	93.8
Labour onset		
Spontaneous	303	31.8
Induced	534	56.1
No labour	105	11
Not stated	10	1.1
Delivery type		
Vaginal	577	60.6
Forceps	16	1.7
Ventouse	15	1.6
Vaginal breech	181	19
Caesarean	151	15.9
Other	8	0.8
Not stated	4	0.4
Antepartum stillbirth		
Yes	864	90.8
No	88	9.2
Baby gender		
Male	465	48.8
Female	476	50
Indeterminate	4	0.4
Not stated	7	0.7
Unexplained stillbirth		
Yes	469	49.3
No	483	50.7
Spontaneous onset of labour		
Yes	303	31.8
No	649	68.2
Weight < 10th Percentile**		
Yes	217	22.8
No	735	77.2
Post mortem performed		
Yes	393	41.3
No	559	58.7
Gestational age (weeks)		
20–24	228	24
25–29	178	18.7
30–36	259	27.2
≥37	287	30.1

*Data for these variables collected from MDC available on 901/952 stillborn babies.

**Calculated using Australian National Birth weight Percentiles [31].

**Table 2:** Relationship of fetal response to spontaneous onset of labour and unexplained fetal death.

	Fetal response *n* = 96 (%)	Absence of fetal response *n* = 856 (%)	Unadjusted odds ratio (95% CI)	Adjusted odds ratio* (95% CI)	*P* value
Spontaneous onset of labour (*n* = 303/952)	65 (67%)	238 (27%)	5.4 (3.5–8.6)	4.4 (2.7–7.2)	< 0.0001
Unexplained death (*n* = 469/952)	15 (16%)	454 (53%)	6.1 (3.5–10.7)	4.5 (2.4 – 8.4)	<0.0001

*Adjusted for: fetal growth restriction < 10th percentile, bleeding during pregnancy, maternal medical conditions, maternal hypertension, fetal or maternal proven infection, and gestational age.

**Table 3:** Relationship of fetal response to cause of death.

		Fetal response		
		Absent	Present	Total

PSANZ classification of cause of death	Perinatal infection	20	18	38
		52.6%	47.4%	100.0%
	Hypertension	58	0	58
		100.0%	.0%	100.0%
	Antepartum haemorrhage	84	3	87
		96.6%	3.4%	100.0%
	Maternal disease	45	1	46
		97.8%	2.2%	100.0%
	Perinatal conditions	76	5	81
		93.8%	6.2%	100.0%
	Hypoxic peripartum death	18	5	23
		78.3%	21.7%	100.0%
	Fetal growth restriction	31	3	34
		91.2%	8.8%	100.0%
	Spontaneous preterm	69	46	115
		60.0%	40.0%	100.0%
	Unexplained antepartum death	454	15	469
		96.8%	3.2%	100.0%
	No obstetric antecedent	1	0	1
		100.0%	.0%	100.0%
	Total	856	96	952
		89.9%	10.1%	100.0%
