# Supplementary material for: Histological Chorioamnionitis Is Increased at Extremes of Gestation in Stillbirth: A Population-Based Study
Source: Infect Dis Obstet Gynecol. 2011 Jul 19;2011:456728. doi: 10.1155/2011/456728 (PMC3140189; doi:10.1155/2011/456728)
Supplement: Supplementary file 1 — Appendix S1: The New South Wales Midwives Data Collection (MDC) is a population-based surveillance system covering all births in NSW public and private hospitals, as well as home births. It encompasses all live births and stillbirths of at least 20 weeks gestation or at least 400 grams birth weight. Appendix S2: The NSW Maternal and Perinatal Committee is a quality assurance committee established under the NSW Health Administration Act 1982 and is privileged under this Act to carry out confidential reviews of both maternal and perinatal deaths. Information available to the POWP at review is forwarded by hospitals and includes a confidential report on perinatal death (Appendix S2), postmortem, and placental pathology reports as well as any other information considered relevant by the local hospital perinatal death review committee. [file 456728.f1.pdf]

Appendix S1: Midwives Data Collection

NSW MIDWIVES DATA COLLECTION

Mother Unit Record No. ☐☐☐☐☐☐☐☐☐☐ Hospital ______________ Code ☐☐☐☐

First Name ☐☐☐☐☐☐☐☐☐☐☐☐ Family Name ☐☐☐☐☐☐☐☐☐☐☐☐☐☐☐

Address ______________________________ Postcode ☐☐☐☐

Mother's birth date ☐☐ / ☐☐ / ☐☐☐☐ (day month year)

Country of birth: Australia ☐ 36 Other ☐

If other, specify ______________ ☐☐☐

Indigenous status: Aboriginal ☐ 1; Torres Strait Islander ☐ 2; Aboriginal and Torres Strait Islander ☐ 3; None of the above ☐ 4

PREVIOUS PREGNANCIES

Previous pregnancy greater than 20 weeks? Yes ☐ 1 No ☐ 0

If no, go to next section.

If yes:

Specify the number of previous pregnancies > 20 weeks ☐☐

Was the last birth by caesarean Yes ☐ 1 No ☐ 0

Total number of previous caesarean sections? ☐

THIS PREGNANCY

Date of LMP ☐☐ / ☐☐ / ☐☐☐☐ (day month year)

Prenatal diagnosis (< 20 weeks gestation) CVS ☐ Amniocentesis ☐

Antenatal care

Duration of pregnancy at first visit (weeks) ☐☐

Not booked ☐

Medical conditions

Diabetes mellitus ☐

Gestational diabetes ☐

Chronic hypertension ☐

Pre-eclampsia ☐

Smoking

Did the mother smoke at all during pregnancy? Yes ☐ 1 No ☐ 0

If yes, how many cigarettes each day on average in the second half of pregnancy?

None ☐ 1; > 10 per day ☐ 2; ≤ 10 per day ☐ 3; Unknown ☐ 4

LABOUR AND DELIVERY

Onset of labour

Spontaneous ☐ 1 Induced ☐ 2

No labour ☐ 3

If labour augmented/ induced (tick 1 or more):

Oxytocins ☐ ARM ☐

Prostaglandins ☐ Other ☐

LABOUR AND DELIVERY

If labour induced, main indication:

Diabetes ☐ 1

Hypertensive disease ☐ 2

Fetal distress ☐ 3

Fetal death ☐ 4

Chorioamnionitis ☐ 5

Blood group isoimmunisation ☐ 6

Prelabour rupture of membranes ☐ 7

Prolonged pregnancy (41+ weeks) ☐ 8

Suspected intrauterine growth restriction ☐ 9

Other ☐ 10

Pain relief/ anaesthetics *(tick 1 or more)*

None ☐ Pudendal ☐

Nitrous oxide ☐ Spinal ☐

IM narcotics ☐ General anaesthetic ☐

Local to perineum ☐

Epidural/caudal ☐ Other ☐

Presentation at birth

Vertex ☐ 1 Face ☐ 3

Breech ☐ 2 Brow ☐ 4

Other ☐ 5

Type of delivery

Normal vaginal ☐ 1 Vacuum extr. ☐ 3

Forceps ☐ 2 Vaginal breech ☐ 4

Caesarean section ☐ 5

If caesarean section, main indication:

Failure to progress

- Cx dilatation unknown ☐ 1
- Cx 3cm dilated or less ☐ 2
- Cx dilated more than 3 cm ☐ 3

Fetal distress ☐ 4

Other ☐ 5

Perineal status

Intact ☐ 1 4th deg. tear ☐ 5

1st deg. tear/graze ☐ 2 Episiotomy ☐ 6

2nd deg. tear ☐ 3 Both tear and episiotomy ☐ 7

3rd deg. tear ☐ 4 Other ☐ 8

Surgical repair of the vagina or perineum? Yes ☐ 1 No ☐ 0

BABY

Place of birth

Hospital theatre/delivery suite ☐ 1

Birth centre ☐ 2

Planned birth centre/delivery suite birth ☐ 3

Planned homebirth ☐ 4

Planned homebirth/hospital admission ☐ 5

Born before arrival ☐ 6

Unit Record No. ☐☐☐☐☐☐☐☐☐☐

Birth date: ☐☐ / ☐☐ / ☐☐☐☐ (day month year)

Sex: M ☐ 1 F ☐ 2 Indet. ☐ 3

Plurality: Single ☐ 1 Multiple ☐ 2

If multiple, total number ☐

If multiple birth, specify baby number ☐

Birthweight (grams) ☐☐☐☐

Estimated gestational age ☐☐

Apgar ☐☐ 1 min ☐☐ 5 min

Resuscitation of baby *(tick 1 or more)*

None ☐ 1 IPPR : bag + mask ☐ 4

Suction ☐ 2 Intubation + IPPR ☐ 5

O2 therapy ☐ 3 External cardiac massage + ventilation ☐ 6

Other ☐ 7

POSTNATAL CARE - BABY

Birth defect? Yes ☐ 1 No ☐ 0

If yes, specify:

Admitted to NICU? Yes ☐ 1 No ☐ 0

Admitted to SCN? Yes ☐ 1 No ☐ 0

If yes, observation only? Yes ☐ 1 No ☐ 0

If admitted to SCN/NICU:

Was a birth defect the main reason for admission? Yes ☐ 1 No ☐ 0

DISCHARGE STATUS - MOTHER AND BABY

Mother

Discharged ☐ 1

Transferred ☐ 2

Died ☐ 3

Baby

Discharged ☐ 1

Transferred ☐ 2

Stillbirth ☐ 3

Neonatal death ☐ 4

Transferred and died ☐ 5

Baby's date of discharge or transfer ☐☐ / ☐☐ / ☐☐☐☐ (day month year)

Hospital transferred to: ______________

If baby died, date of death ☐☐ / ☐☐ / ☐☐☐☐ (day month year)

Signature of midwife at discharge

Health Department Copy

Please complete and forward to: NSW Midwives Data Collection
Patient Data Management Unit, Level 6
Locked Bag 961, North Sydney, NSW 2059

Appendix S2: Confidential Report on Perinatal Death

NSW MATERNAL AND PERINATAL COMMITTEE
CONFIDENTIAL REPORT ON PERINATAL DEATH

Office use: Reference No.:

HOSPITAL: ______________________

MOTHER DETAILS:

Mother's name ______________________

Address: ______________________

____

Medical Record No.: ☐☐☐☐☐☐☐☐☐☐

BABY DETAILS:

Baby's name: ______________________

Type of perinatal death: Stillbirth ☐ Neonatal death ☐

Date of birth/ stillbirth: / /

If liveborn: Date of death: / /

Age at death: day(s) hour(s)

Medical Record No.: ☐☐☐☐☐☐☐☐☐☐

For STILLBIRTHS complete Part A and for NEONATAL DEATHS complete Parts A and B

PART A

1. Was a postmortem examination carried out?

Yes ☐ No ☐ Unknown ☐

If yes, please include a copy of the report.

2. Was histopathological examination of the placenta carried out?

Yes ☐ No ☐ Unknown ☐

If yes, please include a copy of the report.

If no: Placental weight: ☐☐☐ grams

Describe the placental appearance:

____

____

3. Is this baby one of a multiple pregnancy (twin, triplet etc)?

Yes ☐ No ☐ Unknown ☐

If yes: Number of babies ☐☐

Birth order ☐☐

4. Bleeding during pregnancy?

Yes ☐ No ☐ Unknown ☐

If yes: Threatened miscarriage ☐
Placental abruption ☐
Placenta praevia ☐
Vasa praevia ☐
Undetermined ☐
Other ☐

If other, specify:

____

5. Was hypertension present?

Yes ☐ No ☐ Unknown ☐

If yes: Chronic hypertension:
Essential ☐
Secondary eg renal disease ☐
Unspecified ☐
Gestational hypertension ☐
Pre-eclampsia ☐
Chronic + superimposed pre-eclampsia ☐
Unspecified ☐

6. Any other maternal diseases present in pregnancy?

Yes ☐ No ☐ Unknown ☐

If yes: Maternal injury:
Accidental ☐
Non-accidental ☐
Diabetes/gestational diabetes ☐
Sepsis ☐
Other ☐

If other, specify:

____

7. Was the death an unexplained antepartum death?

Yes ☐ No ☐ Unknown ☐

8. When did the death occur?

Before the onset of labour ☐
During labour ☐
Before birth, unknown time ☐
After birth ☐

9. Was there fetal growth restriction (weight less than 10th percentile)?

Yes ☐ No ☐ Unknown ☐

Note: for stillbirths who died before the onset of labour, serial U/S evidence of FGR is required.

10. Was there spontaneous preterm delivery (less than 37 weeks)?

Yes ☐ No ☐ Unknown ☐

If yes, what was the duration of rupture of membranes prior to delivery?

Less than 24 hours ☐
24 hours or more ☐
Unknown ☐

11. Was there intrapartum asphyxia?

Yes ☐ No ☐ Unknown ☐

12. Gestational age: ☐☐ weeks

13. Birthweight: ☐☐☐☐ grams

14. Sex: Male ☐
Female ☐
Indeterminate ☐

15. Onset of labour:

Spontaneous ☐
Induced ☐
No labour ☐

16. Type of delivery:

Normal vaginal delivery ☐
Breech delivery ☐
Caesarean section ☐
Forceps delivery ☐
Ventouse delivery ☐
Other delivery ☐

If other delivery, specify:

____

PART A (continued)

17. Were there cord complications?
Yes ☐ No ☐ Unknown ☐
If yes, describe:

18. Was a major fetal abnormality present?
Yes ☐ No ☐ Unknown ☐
If yes, describe:

19. Was chorioamnionitis present?
Yes ☐ No ☐ Unknown ☐
If yes, diagnosis was:
- Pathological ☐
- Clinical ☐

If yes, specify organism:

20. Infant/ fetal infection?
Yes ☐ No ☐ Unknown ☐
If yes:
- Streptococcus Group B ☐
- E Coli ☐
- Listeria monocytogenes ☐
- Cytomegalovirus ☐
- Parvovirus ☐
- Herpes simplex virus ☐
- Rubella virus ☐
- Toxoplasma ☐
- Syphilis ☐
- Other ☐

If other, specify:

21. Other conditions?
Yes ☐ No ☐ Unknown ☐
If yes:
- Twin-to-twin transfusion ☐
- Fetomaternal haemorrhage ☐
- Uterine abnormality ☐
- Birth trauma ☐
- Haemolytic disease ☐
- Idiopathic hydrops ☐
- Drug dependence/abuse ☐
- Termination of pregnancy ☐
- Other ☐

If other, specify:

22. Classification of obstetric cause of death (see Attachment)
☐

PART B (Neonatal deaths only)

THE MAIN CAUSE OF DEATH (tick one):

1. **Congenital abnormality** ☐
2. **Extreme prematurity** ☐
   If yes, was resuscitation carried out?
   Yes ☐ No ☐ Unknown ☐
3. **Cardio-respiratory**
   - Hyaline membrane disease ☐
   - Meconium aspiration syndrome ☐
   - Primary persistent pulmonary hypertension ☐
   - Pulmonary hypoplasia ☐
   - Chronic neonatal lung disease ☐
   - Other ☐

   If other, specify:
4. **Infection**
   - Congenital bacterial ☐
   - Acquired bacterial ☐
   - Congenital viral ☐
   - Acquired viral ☐
   - Protozoal eg Toxoplasma ☐
   - Spirochaetal eg Syphilis ☐
   - Fungal ☐
   - Other ☐

   If other, specify:
5. **Neurological**
   - Hypoxic ischaemic encephalopathy/ perinatal asphyxia ☐
   - Intracranial haemorrhage ☐
   - Other ☐

   If other, specify:
6. **Gastrointestinal**
   - Necrotising enterocolitis ☐
   - Other ☐

   If other, specify:
7. **Other**
   - SIDS:
     - Consistent with SIDS ☐
     - Possible SIDS ☐
   - Multisystem failure (only if unknown primary cause or trigger event ☐
   - Trauma ☐
   - Undetermined ☐
   - Other ☐

   If other, specify:

MAIN CAUSE OF DEATH AND RELEVANT FACTORS

Main cause of death as determined by hospital perinatal death review committee

Relevant factors:

Antenatal:

Intrapartum:

Postpartum:

Form completed by:
Name:

Date: / /

DEFINITION: FETAL GROWTH RESTRICTION

Less than the 10th percentile for gestation.

Gestation (weeks)	Weight (grams) 10th percentile Male	Female
22	400	400
23	500	470
24	520	540
25	620	620
26	720	680
27	740	730
28	850	760
29	950	890
30	1080	1045
31	1310	1140
32	1400	1340
33	1640	1520
34	1840	1760
35	2110	2030
36	2320	2220
37	2550	2430
38	2780	2660
39	2940	2820
40	3070	2950
41	3180	3050
42	3210	3080
43	3080	2950
44	3050	2930

Source: Roberts CL, Lancaster PAL. Australian national birthweight percentiles by gestational age. *Med J Aust* 1999; 170: 114-118.

Australia and New Zealand Antecendent Classification of Perinatal Mortality

1. **Congenital abnormality**
   (including termination of pregnancy for congenital abnormality)
   - 1.1 Central nervous system
   - 1.2 Cardiovascular system
   - 1.3 Urinary tract
   - 1.4 Gastrointestinal tract
   - 1.5 Chromosmal
   - 1.6 Metabolic
   - 1.7 Multiple
   - 1.8 Other
   - 1.9 Unspecified
2. **Perinatal infection**
   - 2.1 Bacterial
     - 2.11 Group B Streptococcus
     - 2.12 E Coli
     - 2.13 Listeria Monocytogenes
     - 2.18 Other bacterial
     - 2.19 Unspecified bacterial
   - 2.2 Viral
     - 2.21 Cytomegalovirus
     - 2.22 Parvovirus
     - 2.23 Herpes simplex virus
     - 2.24 Rubella virus
     - 2.28 Other viral
     - 2.29 Unspecified viral
   - 2.3 Protozoal eg Toxoplasma
   - 2.4 Spirochaetal eg Syphilis
   - 2.5 Fungal
   - 2.6 Other
   - 2.7 Unspecified organism
3. **Hypertension**
   - 3.1 Chronic hypertension: essential
   - 3.2 Chronic hypertension: secondary eg renal disease
   - 3.3 Chronic hypertension: unspecified
   - 3.4 Gestational hypertension
   - 3.5 Pre-eclampsia
   - 3.6 Pre-eclampsia superimposed on pre-existing hypertension
   - 3.7 Unspecified hypertension
4. **Antepartum haemorrhage**
   - 4.1 Placental abruption
   - 4.2 Placenta praevia
   - 4.3 Vasa praevia
   - 4.8 Other APH
   - 4.9 APH of undertermined origin
5. **Maternal disease**
   - 5.1 Termination of pregnancy (other than for fetal abnormality)
   - 5.2 Diabetes / gestational diabetes
   - 5.3 Maternal injury
     - 5.31 Accidental
     - 5.32 Non-accidental
   - 5.4 Maternal sepsis
   - 5.5 Other maternal conditions eg Lupus obstetric syndrome
6. **Specific perinatal conditions**
   - 6.1 Twin-to-twin transfusion
   - 6.2 Fetomaternal haemorrhage
   - 6.3 Antepartum cord complications
   - 6.4 Uterine abnormality
   - 6.5 Birth trauma (typically >24 weeks or > 600 grams)
   - 6.6 Haemolytic disease
   - 6.7 Idiopathic hydrops
   - 6.8 Other
7. **Hypoxic peripartum death (typically > 24 weeks or > 600 grams)**
   - 7.1 With intrapartum complications
     - 7.11 Uterine rupture
     - 7.12 Cord prolapse
     - 7.13 Shoulder dystocia
     - 7.18 Other
   - 7.2 No intrapartum complications
   - 7.9 Unspecified hypoxic peripartum death
8. **Fetal growth restriction (FGR)**
   - 8.1 With evidence of uteroplacental insufficiency eg significant infarction, acute atherosis, maternal vascular thrombosis or maternal floor infarction
   - 8.2 With chronic villitis
   - 8.3 Without the above placental pathology
   - 8.4 No examination of placenta
   - 8.9 Unspecified FGR or not known whether placenta examined
9. **Spontaneous preterm**
   - 9.1 Spontaneous preterm with intact membranes, or membrane rupture less than 24 hours before delivery,
     - 9.11 with chorioamnionitis
     - 9.12 without chorioamnionitis
     - 9.13 no examination of the placenta
     - 9.19 unspecified or not known whether placenta examined
   - 9.2 Spontaneous preterm with membrane rupture >= 24 hours before delivery,
     - 9.21 with chorioamnionitis
     - 9.22 without chorioamnionitis
     - 9.23 no examination of the placenta
     - 9.29 unspecified or not known whether placenta examined
   - 9.3 Spontaneous preterm with membrane rupture of unknown duration before delivery,
     - 9.31 with chorioamnionitis
     - 9.32 without chorioamnionitis
     - 9.33 no examination of the placenta
     - 9.39 unspecified or not known whether placenta examined
10. **Unexplained antepartum death**
    - 10.1 With evidence of uteroplacental insufficiency eg significant infarction, acture atherosis, maternal vascular thrombosis or maternal floor infarction.
    - 10.2 With chronic villitis
    - 10.3 Without the above placental pathology
    - 10.4 No examination of placenta
    - 10.9 Unspecified unexplained antepartum death or not known whether placenta examined.
11. **No obstetric antecendent**
    - 11.1 SIDS
      - 11.11 Consistent with SIDS
      - 11.12 Possible SIDS
    - 11.2 Postnatally acquired infection
    - 11.3 Accidental asphyxiation
    - 11.4 Other accident, poisoning or violence (postnatal)
    - 11.8 Other
    - 11.9 Unknown / Unexplained
